# Evaluation of treatment response and symptom progression in 400 patients with visual snow syndrome

**DOI:** 10.1136/bjophthalmol-2020-318653

**Published:** 2021-10-16

**Authors:** Francesca Puledda, Nicolas Vandenbussche, David Moreno-Ajona, Ozan Eren, Christoph Schankin, Peter J Goadsby

**Affiliations:** 1 Headache Group, Wolfson CARD, Institute of Psychiatry, Psychology & Neuroscience, King's College London, London, UK; 2 NIHR-Wellcome Trust King’s Clinical Research Facility, SLaM Biomedical Research Centre, King’s College Hospital, NIHR, London, UK; 3 Department of Neurology, University Hospital Ghent, Ghent, Belgium; 4 Neurology, University Hospital LMU Munich, Munchen, Germany; 5 Neurology, University of Bern, Bern, Switzerland; 6 Neurology, University of California Los Angeles, Los Angeles, California, USA

**Keywords:** visual pathway, treatment other

## Abstract

**Aims:**

To gather information on useful medications to treat visual snow syndrome (VSS) as well as to validate an instrument to assess its clinical severity and the course of the disorder over time.

**Methods:**

Four hundred patients with VSS were included in this web-based prospective questionnaire study. All subjects completed a treatment questionnaire and a clinical diary. The first allowed evaluation of the effects of previous medications on visual snow, while the second measured VSS symptoms daily over the course of 30 days.

**Results:**

Patients commonly reported previous use of medications such as antidepressants, antiepileptics, antibiotics and benzodiazepines. However, none of these drug classes was beneficial for the majority of patients. Recreational drugs and alcohol worsened visual snow symptoms in several reports. Vitamins and benzodiazepines had high therapeutic ratios, although in most cases they did not change the course of VSS.

The monthly diary confirmed that the static in VSS is a consistent symptom over time. It also showed that indoor and fluorescent lights have a worse effect on symptoms when compared with natural outdoor lighting.

**Conclusions:**

The study confirms clinical experience that medications are generally ineffective in VSS, with the exception of vitamins and perhaps benzodiazepines, which could be beneficial in some patients. The 30-day diary represents a useful tool to measure symptom progression over time, which could be used in future trials on VSS.

## Introduction

Visual snow (VS) is a primary neurological disorder characterised by the presence of a continuous, pan-field visual disturbance described as small flickering dots, typically of black and white colour.^1^ This phenomenon is termed ‘static’ or ‘snow’ and is unremitting. In addition to the snow, patients with VS syndrome (VSS) present two or more of the following categories of visual symptoms: palinopsia, enhanced entoptic phenomena, photophobia and nyctalopia.^2^ VS was first described as a separate entity only recently^3 4^; however, its increased recognition has led to a growing body of the literature on the subject and has allowed for the condition to be included in the most recent appendix criteria of the International Classification of Headache Disorders.^5^

A recent web-based survey has shown that VSS typically starts in early life and is more troublesome when associated with migraine and tinnitus.^6^ Further evidence from functional brain imaging, spectroscopy and neurophysiology has uncovered functional and anatomical changes in the extrastriate visual cortex in VSS.^7–11^ Even in light of these recent advances, much is still to be understood about the underlying biology of VSS^12^ as well as its optimal management. Importantly, the majority of medications that are commonly used in clinical practice have shown little or no benefit for affected patients.^13^ Another difficulty in the clinical approach to VSS relates to the lack of availability of an instrument to measure its severity and its changes over time.

In this study, our main objectives were to gather information on drug categories that might be useful or harmful in VSS and to define an objective measure of clinical severity. We were also interested in studying the course of the disorder and its possible changes over time or following exposure to different light environments. For this purpose, we developed two distinct questionnaires aimed at assessing VSS. The first, a retrospective ‘treatment questionnaire’, was used to evaluate the effects of previous medications on the main symptoms of VS. The second, a prospective ‘30-day diary’, represented a clinical evaluation of VSS symptoms and was designed to be used on a daily basis over the course of 30 days.

## Methods

### Participant selection and recruitment

The study was advertised on the website of Eye On Vision (http://www.eyeonvision.org/), a patient self-help group for VS, from April 2016 onwards. Most of the patients involved in the study approached our group through a dedicated research email, which they could find on the website. A smaller number of patients had contacted the researchers individually asking to be involved in research and were redirected to the website.

The clinical diagnosis of VSS was evaluated by one of us (FP) through an online survey available on the Eye On Vision website and detailed in table 2 of our previous publication^6^ as well as in the online supplemental material. Further personalised questions were used on a case-by-case basis if the diagnosis was unclear; these clarifications referred mostly to the description of the static and to the exclusion of secondary causes of VS (as per point D of the diagnostic criteria). In the large majority of cases, patients spontaneously reported having had previous normal ophthalmological examinations. These were not analyzed individually for each patient; however, previous examinations were always requested when the patient reported a known clinical abnormality or if there was a clinical doubt on possible secondary causes. A good understanding of the English language was a requirement for inclusion in the study.

10.1136/bjophthalmol-2020-318653.supp1 Supplementary data

Patients who fit the criteria for VSS were sent two questionnaires back via email—the treatment questionnaire and the 30-day diary—in Microsoft Word format (Office 365 for Windows 10) as well as a patient information sheet and a brief explanation on how to complete the questionnaires. These were prepared in collaboration with the patient group and are found in the online supplemental material.

The study was approved by the King’s College London Research Ethics Panel (Reference number: LRS-15/16-2500). Patient consent was based on return of at least one of the questionnaires. Data were collected between April 2016 and April 2018.

### Treatment questionnaire

The treatment questionnaire featured a comprehensive list of commonly used medications, including, among others, antiepileptics, antidepressants, benzodiazepines and pain medication. Patients were instructed to select medications that had been used at least once since symptom onset and to mark the effect of each treatment on their VS symptoms. They could mark 0 for no effect, + for improvement and − for worsening. If patients had not tried any medication since the onset of their symptoms, they were instructed to return the questionnaire blank. Patients were given the possibility of adding any medication that was not on the main list, in a row titled ‘Other’. A column next to the molecule name listed are common brand names for each drug. The treatment questionnaire had a final, blank line, asking patients about previous recreational drug use and its effect on VSS symptoms.

Specific information on dosing or prescriptions was not requested. Nonetheless, when patients specified that they had only taken a medication for a short amount of time, further information was gathered on a case-by-case basis. Data on any treatment known to be used at nontherapeutic doses were not included in the final analysis.

Based on their answers to the treatment questionnaire, a variable was created to categorise each patients’ response to previous medication. The response was classified as either an absence of effect, an improvement, a worsening, a mixed response (improvement and worsening with different drugs). Additionally, patients could state whether they never used medication before. Ratios representing improvement and worsening were calculated as the proportion of these two outcomes over the total times the medication was used; they, therefore, indicate the overall proportion of patients reporting an improvement or a worsening in VSSs when using the drug. Only drugs with a minimum of n=20 reported uses are displayed.

For the analysis, drugs with a known action on the central nervous system were categorised into their different sites of action; as an example, antidepressants were subdivided into selective serotonin reuptake inhibitors (SSRIs), tricyclic antidepressants and atypical antidepressants.

A binary logistic regression analysis was run to predict the effect of the independent variables such as age, gender, age at disease onset (computed as 0 for patients who had VSS for as long as they could remember), presence of tinnitus and migraine on the likelihood of developing any form of response to medication (either an improvement or a worsening).

### Thirty-day static diary

The diary required patients to score seven items, aimed at describing different parameters of the static, daily and over the course of 30 days. Each item was presented with a specific ordinal ranking, with higher numbers representing higher clinical severity. An exception was the element ‘static colour’ in which scores were not ranked based on severity, but rather represented different clinical categories. The seven items were namely: static density (range 0–6), speed (range 0–4), surface dependence (ie, visibility on different surfaces—range 0–4), distraction (ie, levels of distraction caused by the static—range 0–4), time course (ie, variation of the static during 24 hours—range 0–4), colour (black and white, transparent, flashing or coloured static—range 0–5) and size (range 0–5). A detailed clinical definition was given for each score value, to best aid patients’ selection.

In order to define changes of the static parameters over time, median values for each item were tabulated across three equal time periods (days 1–10, days 11–20, days 21–30) and subsequently compared with a Friedman test.

The clinical diary also included a one-off question aimed at characterising symptom response to six different environmental light conditions, both external and internal. These were defined as: ‘outdoor: sunny day’, ‘outdoor: cloudy day’, ‘outdoor: rainy day’, ‘indoor (interior light)’, ‘fluorescent lighting’, ‘outdoor: night-time’ and scored individually on a scale from 1 to 7.

Full details of the diary are provided in the online supplemental material.

### Statistical analysis

Data were tabulated in Excel (Office 365 for Windows V.64). Descriptive statistics, χ² analysis for comparisons of categorical variables, regression analysis, Friedman test for comparisons between groups of ordinal variables with repeated measures and Wilcoxon signed-rank test were performed with SPSS Statistics V.26.0 for Windows (IBM, Armonk, New York: IBM). Violin plots and stacked column graphs were performed in GraphPad Prism V.8.0.0 for Windows V.64 (GraphPad Software, San Diego, California USA, www.graphpad.com) and used to visually represent the 30-day diary and treatment questionnaire data, respectively. p<0.05 was considered significant.

### Data availability

The data that support the findings of this study are available from the corresponding author, on reasonable request.

## Results

### Demographic and clinical characteristics of VSS population

The treatment and diary questionnaires were sent to a total of 1061 subjects with VSS. A total of 380 completed treatment questionnaires, and 200 diaries were returned by 400 subjects. Of these, 208 were women and 192 were men. The mean (±SD) age of participants was 31±11 years; 28% of patients were aged between 16 and 25 years, 41% between 26 and 35 years, 17% between 36 and 45 years and 14% were 46 years or older. Mean (±SD) reported age of onset for VSS symptoms was 14±14 years; 139 patients (35%) reported symptoms for as long as they could remember. The majority of participants came from Europe (n=220; 55%) and North America (n=138; 35%), with a smaller percentage from Australasia (n=16; 4%), Asia (n=13; 3%), Central and South America (n=11; 3%) and Africa (n=2; 0.5%).

The most commonly reported types of static were, in descending order: black and white (n=235; 59%), transparent (n=201; 50%), flashing (n=170; 43%) and coloured (n=155; 39%). With regards to the additional visual symptoms of VSS, floaters (n=348; 87%), afterimages (n=327; 82%) and photophobia (n=325; 81%) were the most common, with patients reporting an average of 6±2 total number of associated symptoms out of eight.

Migraine comorbidity was present in a total of n=266 of the n=362 subjects who were directly queried on it (73%); of these, n=70 reported aura (26%). A total of n=316 (79%) subjects had concomitant tinnitus.

### Results of the treatment questionnaire

**Figure 1:**
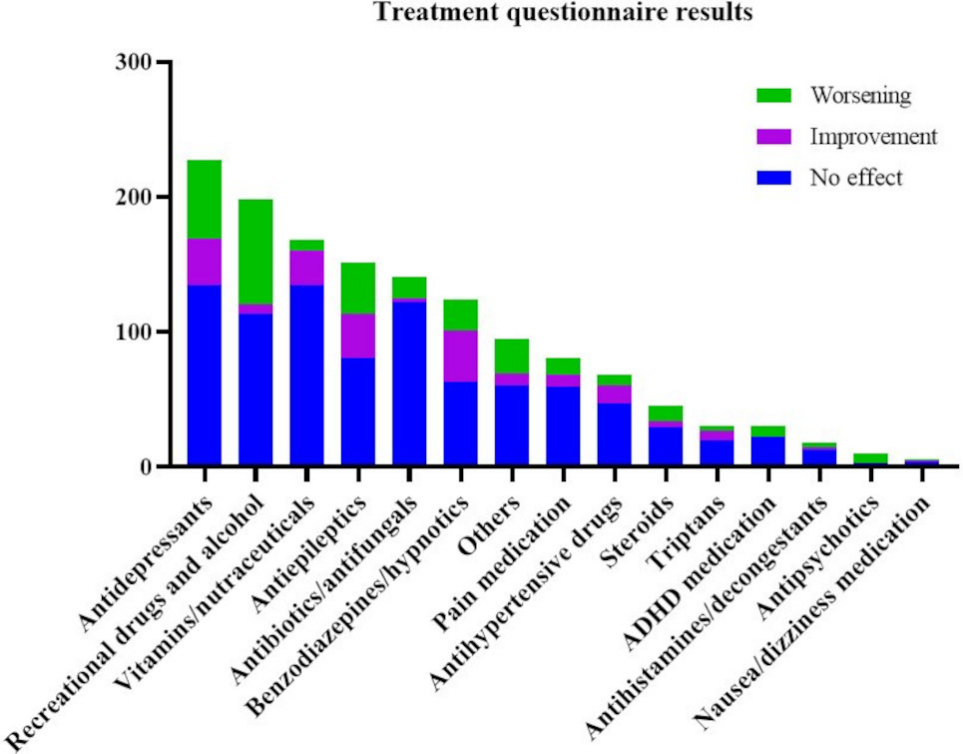
Results from the n=380 treatment questionnaires. Drug categories are shown by total number of times used, with colours indicating total past worsening (green), improvement (magenta), or absence of effect (blue). Exact values are reported in table 1. ADHD, attention deficit hyperactivity disorder.

Of completed questionnaires (n=380), information was gathered on 154 different types of pharmaceutical and nutraceutical compounds, including caffeine, as well as different recreational drugs (n=16), including alcohol (figure 1). Of note, reports on alcohol effects were unprompted, unlike the use of illicit drugs. Table 1 shows the main categories of drugs for which previous use was reported, the number of times each one caused an improvement, a worsening or an absence of effect and its therapeutic ratio or the proportion between the total number of reported improvements and the total number of reported worsenings. This last measure is only shown for drug categories for which the total data points of reported improvement and worsening summed to greater than or equal to 20, as it was not deemed reliable for a smaller number of observations.

**Table 1:** Results of n=380 treatment questionnaires in visual snow syndrome

Drug category	Total times used N	Total times with no effect n (%)	Total times with improvement n (%)	Total times with worsening n (%)	Therapeutic ratio
Antidepressants	**227**	134 (59%)	35 (15%)	58 (26%)	0.6
SSRIs	139	86 (62%)	19 (14%)	34 (24%)	0.6
Tricyclics	61	35 (57%)	12 (20%)	14 (23%)	0.9
Atypical	27	13 (48%)	4 (15%)	10 (37%)	/
Recreational drugs and alcohol	**198**	113 (57%)	7 (4%)	78 (39%)	0.1
Cannabis	99	66 (67%)	1 (1%)	32 (32%)	0.03
Hallucinogens (LSD, psilocybin)	33	17 (52%)	3 (9%)	13 (39%)	/
Stimulants (cocaine, ecstasy)	30	22 (73%)	/	8 (27%)	/
Alcohol	16	/	3 (19%)	13 (81%)	/
Vitamins/nutraceuticals	**168**	134 (80%)	26 (15%)	8 (5%)	3.3
Antiepileptics	**151**	81 (54%)	32 (21%)	38 (25%)	0.8
Topiramate	40	22 (55%)	7 (18%)	11 (28%)	/
Lamotrigine	34	15 (44%)	7 (21%)	12 (35%)	/
Gabapentin	31	19 (61%)	6 (19%)	6 (19%)	/
Valproic acid	19	14 (74%)	2 (11%)	3 (16%)	/
Pregabalin	17	7 (41%)	7 (41%)	3 (18%)	/
Antibiotics/antifungals	**141**	122 (87%)	3 (2%)	16 (11%)	/
Benzodiazepines/hypnotics	**124**	63 (51%)	38 (31%)	23 (19%)	1.7
Others (caffeine, contraceptives, etc.)	**93**	59 (63%)	9 (10%)	26 (28%)	0.3
Pain medication	**81**	59 (73%)	9 (11%)	13 (16%)	0.7
NSAIDs	44	34 (77%)	5 (11%)	5 (11%)	/
Paracetamol/acetaminophen	21	18 (86%)	2 (10%)	1 (5%)	/
Opioids	16	7 (44%)	2 (13%)	7 (44%)	/
Antihypertensive drugs	**68**	47 (69%)	13 (19%)	8 (12%)	1.6
Steroids	**45**	29 (64%)	5 (11%)	11 (24%)	/
Triptans	**30**	20 (67%)	7 (23%)	3 (10%)	/
ADHD medication (amphetamine-type, atomoxetine, methylphenidate)	**26**	12 (46%)	4 (15%)	10 (38%)	/
Antihistamines/decongestants	**18**	13 (72%)	1 (6%)	4 (22%)	/
Antipsychotics	**10**	3 (30%)	/	7 (70%)	/
Nausea/dizziness medication	**8**	4 (50%)	1 (13%)	1 (13%)	/

Drug categories are ordered by total reported number of times used in the past. Therapeutic ratio is measured as number of reported improvements/number of reported worsenings; it is shown only for drugs for which number of reported improvement + number of reported worsening ≥20.

ADHD, attention deficit hyperactivity disorder; LSD, lysergic acid diethylamide; NSAIDs, non-steroidal anti-inflammatory drugs; SSRIs, selective serotonin reuptake inhibitors.

The most commonly reported medications used in the past were antidepressants, followed by antiepileptics, antibiotics and benzodiazepines. Recreational drugs and vitamins had also been frequently used. The highest therapeutic ratios were seen for vitamins and nutraceuticals, although these conversely had no effect on symptoms in 80% of cases as well as benzodiazepines and hypnotics.

Table 2 shows the top categories of medications most commonly associated with an absence of effect, an improvement or a worsening. The table also shows improvement and worsening ratios (see methods section for description).

**Table 2:** Top eight medications and/or drug categories by percentage of no effect, improvement and worsening, with respective ratios (number of reported improvements/total times used)

	Total times used (n)	No effect (%)	Improvement (%; ratio)	Worsening (%; ratio)
Drug category by no effect (%)				
Antibiotics/antifungals	141	87%		
Paracetamol/acetaminophen	21	86%		
Vitamins/nutraceuticals	168	80%		
NSAIDs	44	77%		
Beta-blockers	48	69%		
Triptans	30	67%		
Steroids	45	64%		
SSRIs	139	62%		
Drug category by improvement (%)				
Benzodiazepines/hypnotics	124	51%	31%; 0.3	
Triptans	30	67%	23%; 0.2	
Lamotrigine	34	44%	21%; 0.2	
Tricyclic antidepressants	61	57%	20%; 0.2	
Gabapentin	31	61%	19%; 0.2	
Beta-blockers	48	69%	19%; 0.2	
Topiramate	40	55%	18%; 0.2	
Vitamins/nutraceuticals	168	80%	15%; 0.2	
Drug category by worsening (%)				
Recreational drugs and alcohol	198	57%		39%; 0.4
ADHD medication	26	46%		38%; 0.4
Atypical antidepressants	27	48%		37%; 0.4
Lamotrigine	34	44%		35%; 0.4
Topiramate	40	55%		28%; 0.3
SSRIs	139	62%		24%; 0.3
Steroids	45	64%		24%; 0.2
Tricyclic antidepressants	61	57%		23%; 0.2

Only values for drugs having been used a minimum of n=20 times are displayed.

ADHD, attention deficit hyperactivity disorder; NSAIDs, nonsteroidal anti-inflammatory drugs; SSRIs, selective serotonin reuptake inhibitors.

### Individual response to medication

A total of n=54 subjects had no exposure to previous medication since the onset of VSS and could, therefore, not be taken into account for medication response. Three-hundred and twenty-six patients, on the contrary, had used some form of medication in the past, either for VSS or for other purposes. Of these patients, the large majority (n=158; p<0.001) reported no effect on VSS to any of the medications that they had used. An almost equal number had either some form of improvement (n=61) or worsening (n=63) with at least one medication, and 44 had a mixed effect depending on the specific drug.

A binary logistic regression analysis to predict the effect of clinical VSS variables on the likelihood of having any kind of response to medication showed a significant association between older age at onset (OR 1.02; 95% CI 1.01 to 1.04; p=0.01) and response to previous medication use (online supplemental eTable1).

### Thirty-day diary results

**Figure 2:**
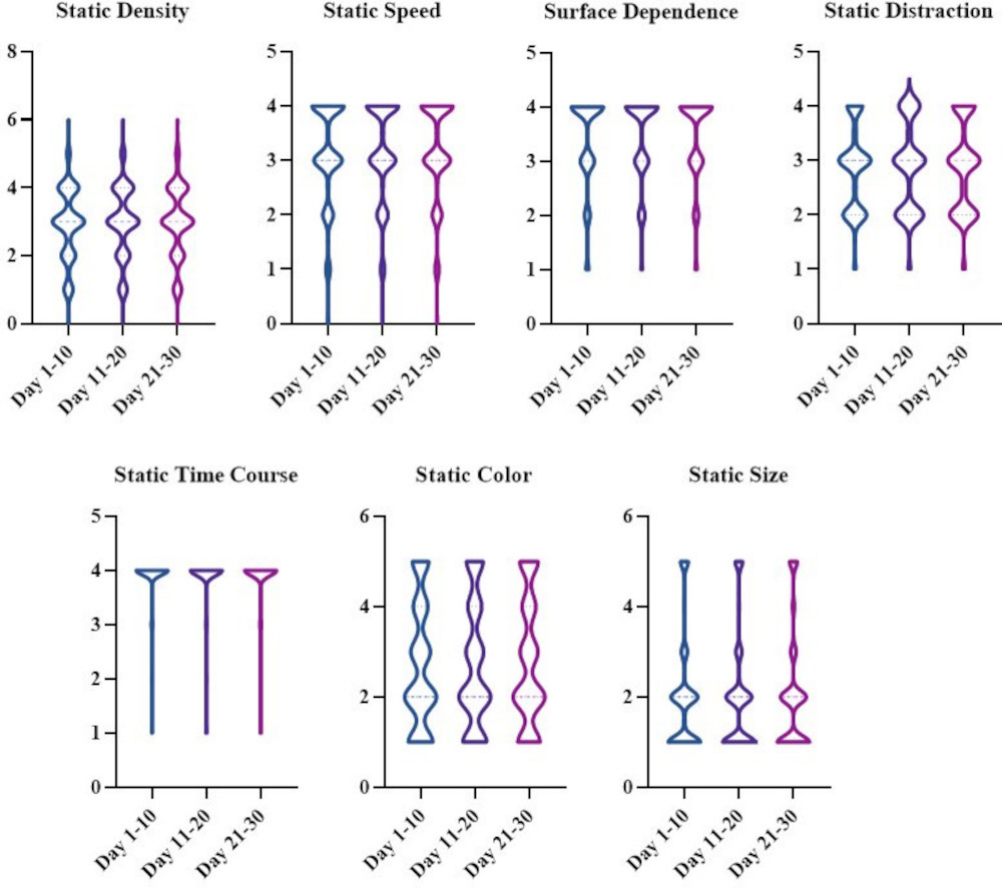
Violin plots showing distribution of the seven static parameters (reported as median and interquartile ranges) of the 30-day diary. Ranges for each value can be found in table 3 and in the online supplemental material.

The median and interquartile ranges for the diary parameters of static density, speed, surface dependence, distraction, time course, colour and size are reported in table 3 (upper panel) and in figure 2. A Friedman test for repeated measures showed no significant variation across the three 10-day periods: 1–10, 11–20 and 21–30, for all parameters.

**Table 3:** Results from the 30-day diary

Static parameters	Range	Median; IQR over 30 days	Median; IQR days 1–10	Median; IQR days 11–20	Median; IQR days 21–30	P value
Static density	0–6	3; 2–4	3; 2–4	3; 2–4	3; 2–4	0.1
Static speed	0–4	3; 3–4	3; 3–4	3; 3–4	3; 3–4	0.6
Surface dependence	0–4	4; 3–4	4; 3–4	4; 3–4	4; 3–4	0.3
Distraction	0–4	3; 2–3	3; 2–3	3; 2–3	3; 2–3	0.4
Time course	0–4	4; 4–4	4; 4–4	4; 4–4	4; 4–4	0.9
Colour	0–5	2; 2–4	2; 2–4	2; 2–4	2; 2–4	0.05
Static size	0–5	2; 1–2	2; 1–2	2; 1–2	2; 1–2	0.5

Light conditions		Range	Median; IQR	A	B	C	D	E	F
Outdoor: sunny day	A	1–7	5; 3–6	/	0.04	0.2	**<0.001**	**<0.001**	**<0.001**
Outdoor: cloudy day	B	1–7	5; 3–6		/	0.3	**<0.001**	**<0.001**	**<0.001**
Outdoor: rainy day	C	1–7	4; 4–5			/	**<0.001**	**<0.001**	**<0.001**
Indoor (interior light)	D	1–7	3; 2–4				/	0.008	**<0.001**
Fluorescent lighting	E	1–7	3; 2–4					/	**0.004**
Outdoor: night-time	F	1–7	1; 1–3						/

Upper panel shows median and interquartile ranges (IQR) for the seven static parameters in the diary. The right column shows the results of Friedman test for comparison between values of days 1–10, days 11–20 and days 21–30.

Lower panel shows results (expressed as median and IQR) for the reported effects of light conditions on the visual static. Each light condition was scored individually on a scale from 1 to 7. The right side of the table shows p values for Wilcoxon test comparing each light condition to the others, with significant results (p<0.008 following multiple comparisons correction) highlighted in bold.

Responses to lights were available for 134 subjects and are shown in table 3 (lower panel). A Friedman test showed a difference (p<0.001) in the scores given to the different light conditions. To determine which of the parameters was driving this effect, a post hoc analysis with Wilcoxon signed-rank test with Bonferroni correction was conducted, resulting in a significance level set at p<0.008. This analysis showed that the scores given to ‘outdoor night time’ were lower than those of all other conditions (p<0.001), and that outdoor day-time conditions (either sunny, cloudy or rainy) were all attributed higher scores with respect to indoor (fluorescent or interior light) or night-time light conditions (table 3).

## Discussion

This study represents the first attempt to investigate systematically retrospective medication use in a substantial cohort of patients with VSS. Simultaneously, it provides a prospective recording of symptom variation through time with an objective measure of clinical severity.

### Medical treatment of VS

The data from our treatment questionnaire confirm an aspect that had already been emerging from the VS literature, specifically that common drug classes used in clinical practice are not beneficial for the majority of affected patients. In fact, most drugs that were recorded showed no change on VSS symptoms in most cases, and only a minority of individuals had some kind of medication effect to report. Furthermore, no single drug for which use was recorded at least 10 times had an improvement rate of more than 20%. The single exception was benzodiazepines, the only category showing an improvement ratio of above 0.2. For certain pharmaceutical classes, such as SSRIs, antiepileptics and antibiotics, these results appear particularly reliable, as their previous recorded use lies in the hundreds. It should further be noted that even if the broad category of antihypertensives showed a high therapeutic ratio, this group included a large variety of medications, such as beta-blockers and calcium channel blockers, with very different mechanisms of actions.

Another important inference emerging from the data is that some substances should probably be avoided in patients with VSS. The main example of this is represented by recreational drugs and alcohol, which have shown to worsen symptoms in almost 40% of VSS cases (although reports on alcohol use were unprompted and might, thus, have been skewed towards the recall of a particular effect). Other drug categories that should be used with caution are atypical antidepressants and ADHD medication. Even lamotrigine and topiramate, two antiepileptics that have been anecdotally reported as useful in the past, caused a worsening in up to 35% of cases recorded here, which was higher than their frequency of improvement (21% and 18%, respectively). It is important to note that the reduction of symptoms^14^ and even remission rates found in these previous studies^15^ were never reported for more than five VSS patients.

Interestingly, having any kind of medication response appeared less likely in patients who had an earlier age of onset, as shown by the regression results.

Finally, our results confirm that even if VSS is strongly comorbid with migraine,^4 13 15 16^ most acute pain relief options and even preventive migraine medication—such as tricyclic antidepressants and beta-blockers—appear relatively useless in this condition.

In order to aid clinicians treating patients with VSS, therapeutic ratios have been provided for most drug categories. These ratios are an indirect measure of the overall safety of a specific drug, as they take into account both the improvement and worsening likelihoods. The measure is presented only when the sum of reported improvements and worsening exceeded 20, as this was felt to be the minimum number of observations to allow a meaningful interpretation of the data. Therapeutic ratios show that vitamins and nutraceuticals might represent viable options for patients who are struggling with severe symptoms, given that they mostly cause no harm (80% of the times) and occasionally bring some benefit (15% of cases). However, the overall lack of response of VSS to medical treatment does seem to highlight the importance of exploring nonpharmacological interventions, such as yellow-blue tinted lenses^16^ or neuromodulation, as a means for treating the disorder in the future.

### Symptom recording and environmental influence

The most relevant finding from the monthly diary recording is that the parameters used to measure the visual static in VSS are highly stable through time. Even though the result was expected based on the clinical description of the condition, this was the first time VS symptoms were objectively recorded through time in a large amount of patients.

The diary, therefore, has the potential of being used as a measure of symptom progression in VS, both with and without the full syndrome presentation, and could prove particularly useful in the future to assess individual response to different therapeutic approaches as well as in the development of randomised controlled studies.

The diary data also allowed to pinpoint specific light conditions that can worsen VS symptoms, such as indoor and fluorescent lighting, as opposed to natural outdoor lighting. This information can be used to give relevant advice to affected patients, potentially improving their quality of life.

### Limitations

The main limitation of this study relates to its recruitment methodology. The web-based approach was used to reach a large number of patients, however, this could certainly have biased towards a population that was younger and with more severe symptomatology. This possibility seems to be confirmed by a recent population-based study performed on VS, where a significantly older mean age was reported.^17^ Our population also had low representation of African and Asian countries, and this might have influenced the results as well.

Furthermore, although the study involved patients with the complete VSS, most questions focused solely on aspects of the static, as this is the only symptom that all patients have in common. In the future, it would certainly be useful to expand the evaluation to include other VSS symptoms such as photophobia and entoptic phenomena.

It must further be noted that the VS diary only allows to establish stability of symptoms over a 30-day period, and further longitudinal studies will be needed to confirm this over longer periods. Nonetheless, the authors’ clinical experience from interviewing hundreds of patients over the years is that this indeed represents the most common course of presentation for the condition.

Finally, data on previous treatment use were based solely on patient recollection, and could, therefore, have been subjected to a certain element of bias, particularly with regards to dosing. This aspect was, however, deemed necessary due to the exploratory nature of the questionnaire, and in order to maintain the study as accessible as possible for a broad patient population.

## Conclusions

In conclusion, this study shows that common drug treatments are not useful in the majority of cases of VSS and that recreational drugs can worsen its symptomatology. Vitamins and nutraceuticals could be beneficial in some patients and can be considered particularly safe for the condition. Further studies, ideally randomised controlled trials, are needed in order to understand the full effects of medications such as benzodiazepines on VSS.

The clinical diary allows to assess the variations of visual static objectively. We believe that this instrument will aid research on treatment strategies for VSS in the future, for which there is certainly an unmet and pressing need.

In summary, clinicians who are most likely to encounter VS should be mindful of avoiding certain drugs such as stimulants and atypical antidepressants when possible, as well as recreational substances, which can certainly worsen symptoms. They might also want to counsel patients on the benign and relatively stable nature of the condition. Finally, as nutraceuticals appear harmless and might benefit some patients, they could be considered as a therapeutic option in certain cases.

## Data Availability

Data are available upon reasonable request.
