# Supplementary material for: Evaluation of treatment response and symptom progression in 400 patients with visual snow syndrome
Source: Br J Ophthalmol. 2021 Oct 16;106(9):1318–24. doi: 10.1136/bjophthalmol-2020-318653 (PMC9411880; doi:10.1136/bjophthalmol-2020-318653)
Supplement: Supplementary data [file bjophthalmol-2020-318653supp001.pdf]

An evaluation of treatment response and symptom progression in four hundred patients with visual snow syndrome

Supplementary material

- Clinical criteria for the definition of VSS and online survey used for diagnosis are shown respectively in sections A and B of this supplementary material.
- The treatment questionnaire and the 30-day diary are outlined respectively in sections C and D.
- eTable1 represents the results from the binary logistic regression analysis to predict the effect of clinical VSS variables on the likelihood of having any kind of response to medication.

A) Clinical criteria for the definition of the visual snow syndrome

**A**	**Visual snow: dynamic, continuous, tiny dots in the entire visual field lasting longer than 3 months.**<br>*The dots are usually black/grey on white background and grey/white on black background; however they can also be transparent, white flashing or coloured.*
**B**	**Presence of at least two additional visual symptoms of the four following categories:**<br>(i) Palinopsia. At least one of the following: afterimages or trailing of moving objects.<br>*After images should be different from retinal afterimages, which occur only when staring at a high contrast image and are in complementary colour.*<br>(ii) Enhanced entoptic phenomena. At least one of the following: excessive floaters in both eyes, excessive blue field entoptic phenomenon, self-light of the eye, or spontaneous photopsia.<br>*Entoptic phenomena arise from the structure of the visual system itself. The blue field entoptic phenomenon is described as uncountable little grey/white/black dots or rings shooting over visual field in both eyes when looking at homogeneous bright surfaces, such as the blue sky; self-light of the eye is described as coloured waves or clouds when closing the eyes in the dark; spontaneous photopsia is characterized by bright flashes of light.*<br>(iii) Photophobia.<br>(iv) Nyctalopia.
**C**	**Symptoms are not consistent with typical migraine visual aura.**<br>*As defined by the International Headache Society in the International Classification of Headache Disorders [12].*
**D**	**Symptoms are not better explained by another disorder.**<br>*Normal ophthalmology tests (best corrected visual acuity, dilated fundus exam, visual field and electroretinogram); not caused by previous intake of psychotropic drugs.*

B) Online survey

Name<br>Address<br>Date of Birth (Day/Month/Year)<br>Telephone number
1) Please make a brief statement that you are willing to be contacted for research. This is a European data protection issue. example: "Yes, please keep my contact details and you may contact me for research purposes."
2) Brief description of all symptoms you relate to visual snow syndrome.
3) Date or age when your symptoms started.
4) Visual snow: what type<br>- black and white (i.e. only black dots on white background, white dots on black background)<br>- clear (i.e. colour of the background)<br>- flashing (i.e. always white, brighter than background)<br>- coloured<br>- all of these
5) Other symptoms (please only answer yes or no)<br>- After images<br>- Trailing of images in the vision<br>- Blue field entoptic phenomenon (i.e. white squiggly lines moving pulsating on the blue sky)<br>- Floaters in vision<br>- Coloured clouds or waves with eyes closed<br>- Flashes of light<br>- Impaired night vision<br>- Sensitive to light<br>- Tinnitus
6) Have you ever been diagnosed with migraine or have you had a headache of moderate or severe intensity in the past? (Please answer yes or no)
7) Have you ever taken any illicit drugs in the past?

C) Treatment questionnaire.

Drug name	Brand name (examples)	Effect	Symptom	Please describe effect here
Topiramate	Topamax, Sincronil, Acomicil, Neutop			
Gabapentin	Neurontin, Alpentin, Gabagamma, Dineurin			
Valproic acid	Depakin, Depakote, Epilim, Divalproex, Orfiril			
Lamotrigine	Lamictal, Lamotrix, Lafigin, Lambipol			
Pregabalin	Lyrica, Maertesia, Pagadin			
Other antiepileptics				
Sertraline	Zoloft, Setrax, Serlift, Serenata, Asentra			
Fluoxetine	Prozac, Sarafem, Fluxen, Deprex			
Amitriptyline	Elavil, Laroxyl, Endep			
Nortriptyline	Aventyl, Pamelor			
Other antidepressants				
Lorazepam	Ativan, Temesta, Lorans			
Clonazepam	Klonopin, Rivotril, Clonex			
Alprazolam	Xanax, Zolam, Kalma, Restyl			
Other anxiety or sleep medications				
Sumatriptan	Imigran, Imitrex, Sumavel			
Eletriptan	Relpax			
Other triptans				
Methylprednisolone	Solu-Medrol, Depo-Medrol, Medrol			
Prednisolone	Deltacortril, Deltasone, Pred Forte,			
Other steroids or pain medications				
Doxycycline	Acticlate, Adoxa, Hydrodoxx, Oraxyl			
Sulfamethoxazole and trimethoprim	Bactrim, Septrin, Cotrim			
Amoxicillin	Augmentin, Amoxil, Trimox			
Metronidazole	Flagyl, Gynotran, Noritate, Rozex			
Other antibiotics or antifungals				
Atomoxetine	Strattera, Abretia			
Other ADHD treatments				
Propanolol	Inderal, Innopran			
Verapamil	Calan, Isoptin, Verelan			
Other antihypertensive treatments				
Vitamins and other supplements				
Metoclopramide				
Other				

D) 30-day diary of visual snow.

**A - Static Density**<br>**Check only one**		**B - Static Speed**<br>**Check only one**	
0 - No static.	☐	0 - Not recognizable	☐
1 - Minimal static (very thin distribution of dots). Does not mask vision.	☐	1 - Almost still	☐
2 - Minimal to moderate static (faint distribution of dots). Slight masking of vision.	☐	2 - Slow	☐
3 - Moderate static. Some masking of vision.	☐	3 - Moderate	☐
4 - Strong static. Substantial masking of vision. Still able to see through.	☐	4 - Rapid	☐
5 - Very strong static. Distracting masking of vision. Barely able to see through.	☐		
6 - Extreme static, not able to see through.	☐		

**C - Surface dependence**<br>**Check only one**		**D – Distraction**<br>**Check only one**		**E - Time course**<br>**Check only one**	
0 - Cannot be seen on any surface.	☐	0 - Impossible to notice.	☐	0 - Not present	☐
1 - Can be seen on few surface.	☐	1 - Able to see if concentrating.	☐	1 - Present occasionally (< 8 hrs/d)	☐
2 - Can be seen on many surfaces.	☐	2 - Able to see without concentrating. Can be ignored most of the time.	☐	2 - Present most of the time (8-16 hrs/d)	☐
3 - Can be seen on most, but not all surfaces.	☐	3 - Able to see without concentrating. Can be ignored sometimes.	☐	3 - Present almost always (16-24 hrs/d)	☐
4 - Can be seen on all surfaces.	☐	4 - Able to see without concentrating. Cannot be ignored.	☐	4 - Continuous presence	☐

**F – Colour**<br>**Check only one**		**G – Size**<br>**Check only one**	
1 - Black and white (only black dots on white background or white dots on black background)	☐	1 - Tiny	☐
2 - Transparent (colour of the background)	☐	2 - Small	☐
3 - Flashing (always white, brighter than background)	☐	3 - Medium	☐
4 - Coloured	☐	4 - Large	☐
5 - Other (please explain briefly, try to use singular descriptors or adjectives)_______________________________________	☐	5 - Pixelated	☐

**H - Lighting conditions**<br>**please rate from 1 to 7, with 1 being the worst condition and 7 the best (only rate once)**
Outdoor: sunny day
Outdoor: cloudy day
Outdoor: rainy day
Indoor (interior light only)
Fluorescent lighting
Outdoor: night-time

	Day 1	Day 2	Day 3	Day 4	Day 5	Day 6	Day 7	Day 8	Day 9	Day 10
**Date**										
**A - Static Density**										
**B - Static Speed**										
**C - Surface dependence**										
**D - Distraction**										
**E - Time course**										
**F - Colour**										
**G - Size**										

	Day 11	Day 12	Day 13	Day 14	Day 15	Day 16	Day 17	Day 18	Day 19	Day 20
**Date**										
**A - Static Density**										
**B - Static Speed**										
**C - Surface dependence**										
**D - Distraction**										
**E - Time course**										
**F - Colour**										
**G - Size**										

	Day 21	Day 22	Day 23	Day 24	Day 25	Day 26	Day 27	Day 28	Day 29	Day 30
**Date**										
**A - Static Density**										
**B - Static Speed**										
**C - Surface dependence**										
**D - Distraction**										
**E - Time course**										
**F - Colour**										
**G - Size**										

INSTRUCTIONS: Please score each symptom from A to G, on a daily basis.
Please indicate your start (__/__/20__) and end date (__/__/20__).

eTable 1. Binary logistic regression analysis results for prediction of no response to medication vs. any response to medication. OR = odds ratio; CI = 95% confidence interval.

	OR	CI	*P* value
Age	1.00	0.98 – 1.02	0.89
Male gender	.87	0.54 – 1.41	0.57
Age of onset	1.02	1.01 – 1.04	0.01
Tinnitus	1.31	0.74 – 2.33	0.36
Migraine	1.11	0.64 – 1.93	0.71
